# Supplementary material for: An Outbred Guinea Pig Disease Model for Lassa Fever Using a Host-Adapted Clade III Nigerian Lassa Virus
Source: Viruses. 2023 Mar 17;15(3):769. doi: 10.3390/v15030769 (PMC10052409; doi:10.3390/v15030769)
Supplement: Supplementary file 1 [file viruses-15-00769-s001.zip › viruses-2244650-supplementary.pdf]

Table S1: Whole blood samples collected at specified times post-inoculation were tested for the presence of LASV RNA using a qualitative real-time RT-PCR assay.

Day After Infection	Guinea Pig	Ct	Average Ct
**Day 1**	79	0	0
	80	0	
	81	0	
	82	0	
**Day 3**	83	37.4	36.7
	84	0	
	85	35	
	86	37.7	
**Day 6**	87	32.9	33.2
	88	32.3	
	89	33.1	
	90	34.4	
**Day 9**	91	33.6	31.8
	92	31.8	
	93	30.5	
	94	31.4	
**Day 12**	95	31.7	29.8
	96	28.8	
	97	28.1	
	98	30.4	
**Day 15**	104	33.3	33.3

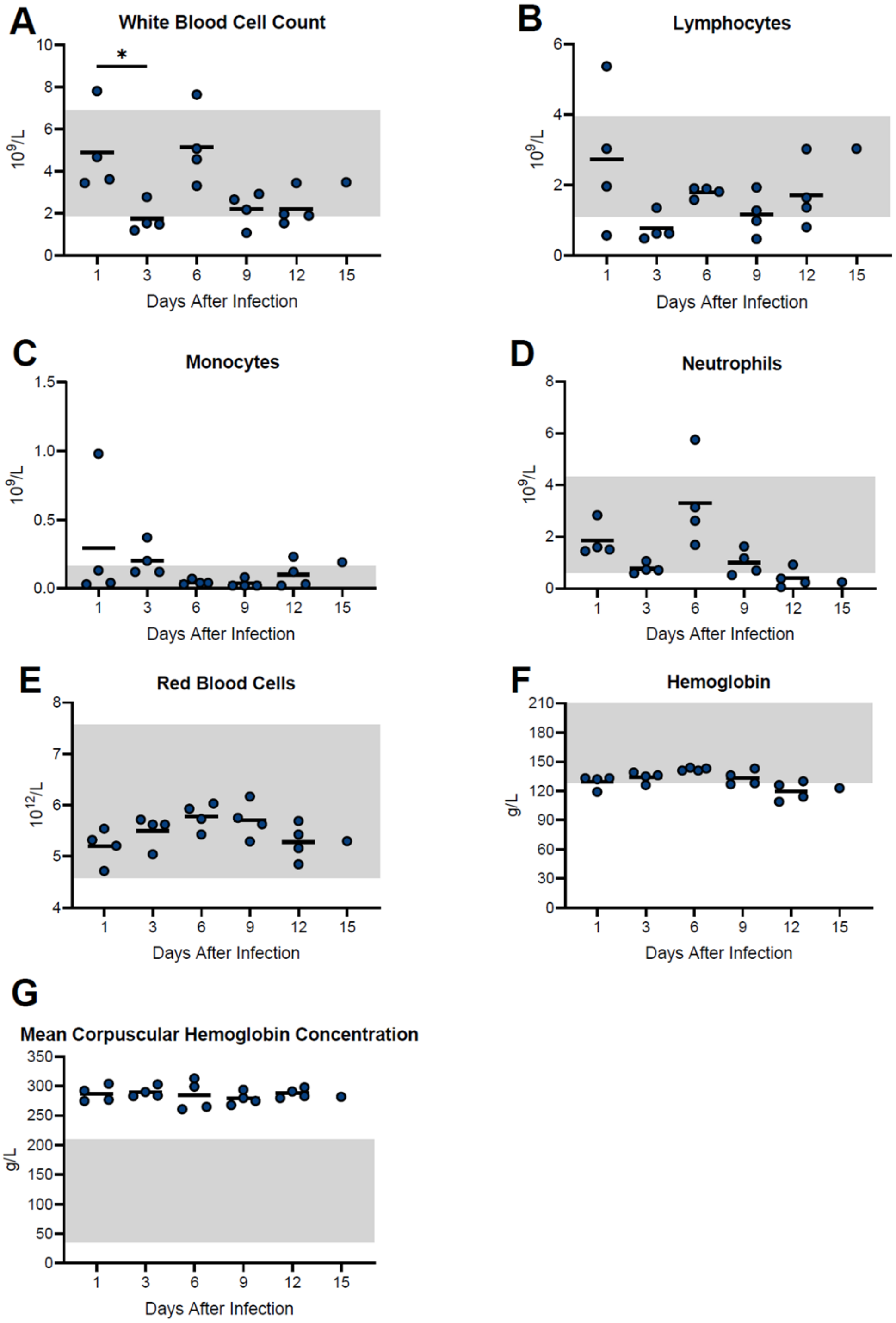

Figure S1: Groups of four animals were euthanized at indicated days post-inoculation and analyzed for hematological parameters. Shown are data for white blood cell count (**A**), lymphocytes (**B**), monocytes (**C**), neutrophils (**D**), red blood cells (**E**), hemoglobin (**F**), and mean corpuscular hemoglobin concentration (**G**). Dots represent individual animal values. bar represents group averages; Significant differences where $p < 0.05$ are indicated by *.

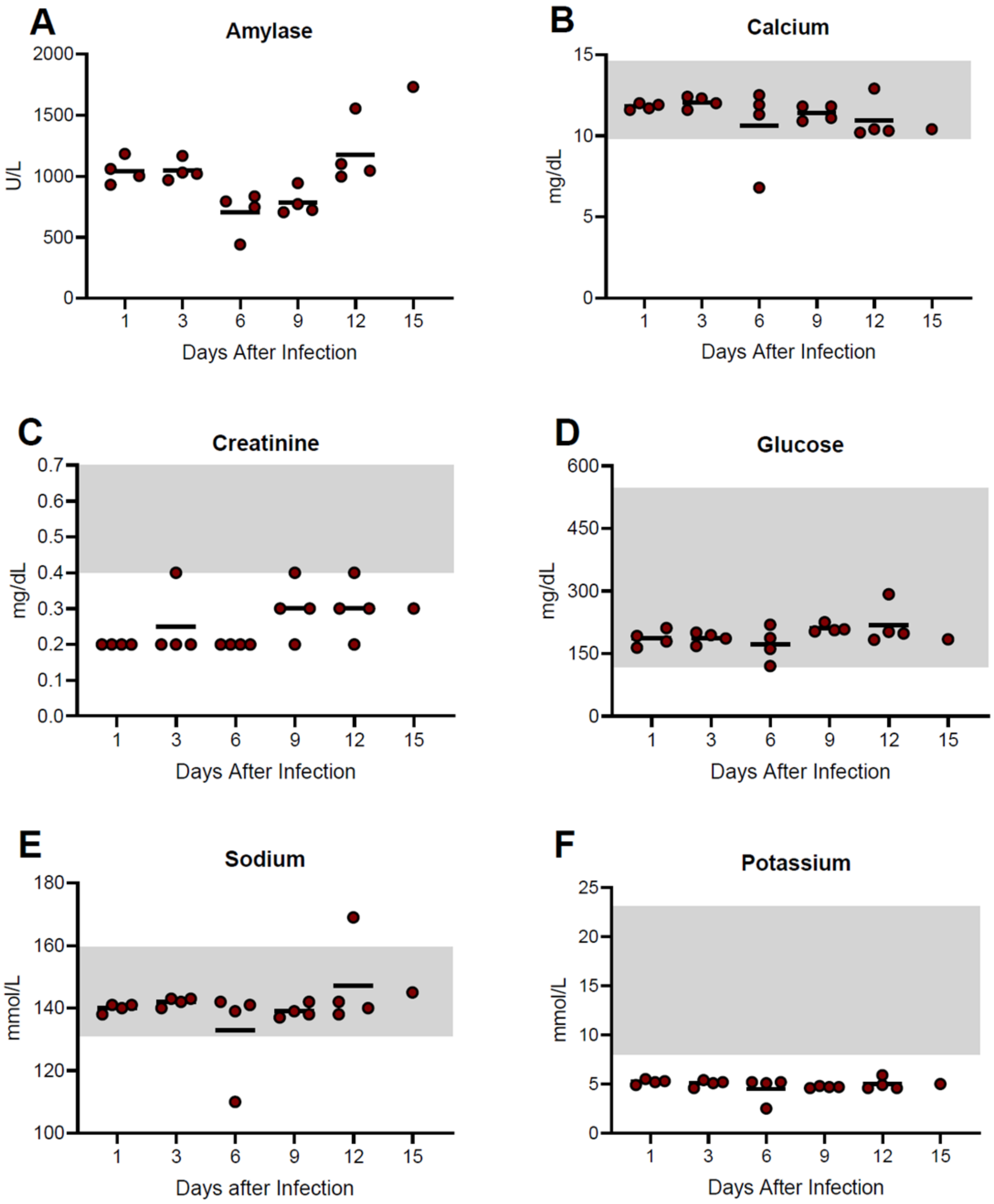

Figure S2: Groups of four animals were euthanized at indicated days post-inoculation and analyzed for biochemical parameters. Shown are data for amylase (**A**), calcium (**B**), creatinine (**C**), glucose (**D**), sodium (**E**), and potassium (**F**). Dots represent individual animal values. bar represents group averages. Where available, normal ranges are indicated by gray shading (www.criver.com, accessed on 8 March 2023).
